# Humanized and Community-Based Nursing for Geriatric Care: Impact, Clinical Contributions, and Implementation Barriers

**DOI:** 10.3390/nursrep15080302

**Published:** 2025-08-18

**Authors:** Viviana Margarita Espinel-Jara, María Ximena Tapia-Paguay, Amparo Paola Tito-Pineda, Eva Consuelo López-Aguilar, Eloy Fernández-Cusimamani

**Affiliations:** 1 Department of Nursing, Faculty of Health Sciences, Universidad Técnica del Norte, Avda. 17 de Julio 5-21, Ibarra 100105, Ecuador; vmespinel@utn.edu.ec (V.M.E.-J.); mxtapia@utn.edu.ec (M.X.T.-P.); aptito@utn.edu.ec (A.P.T.-P.); eclopez@utn.edu.ec (E.C.L.-A.); 2 Department of Crop Sciences and Agroforestry, Faculty of Tropical AgriSciences, Czech University of Life Sciences Prague, Kamýcká 129, Suchdol, 165 00 Prague, Czech Republic

**Keywords:** humanized nursing, community-based nursing, geriatric care, aging in place, implementation barriers

## Abstract

**Background**: As global populations age, the demand for effective and compassionate geriatric care has intensified. Humanized nursing emphasizes empathy and person-centered care, while community-based nursing builds on local resources and networks to support health and well-being. Together, these approaches offer promising strategies for improving care for older adults. This integrative review explores the evolution, clinical contributions, and implementation barriers of these models. **Methods**: A comprehensive literature search was performed using PubMed, CINAHL, Scopus, and Web of Science, targeting peer-reviewed studies, including qualitative and quantitative studies published between 2010 and 2025, that involved adults aged 60 years and older. Inclusion criteria emphasized humanized and community-based nursing interventions while excluding non-nursing and pediatric-focused studies. Quality appraisal was performed using CASP and JBI checklists, and data were thematically synthesized. **Results**: Evidence indicates that these care models significantly improve functional independence and psychosocial well-being and reduce hospital readmissions. For instance, community-based care in Taiwan improved activities of daily living in dementia patients by 15%, while U.S.-based programs reduced depressive symptoms by 30% among Latino older adults. Interdisciplinary, nurse-led interventions in South Korea and Puerto Rico showed a 22% reduction in readmissions and an 85% increase in care access. Despite these benefits, numerous barriers hinder widespread implementation, including workforce shortages, inadequate funding, fragmented healthcare systems, cultural resistance, digital literacy challenges, and policy constraints, particularly in low-resource settings such as the Philippines and Nepal. **Conclusions**: These findings underscore the transformative potential of humanized and community-based nursing while highlighting the need for targeted strategies such as task-shifting, inclusive technologies, and policy reform to advance equitable, sustainable geriatric care globally.

## 1. Introduction

Humanized nursing care is an approach centered on a holistic view of the patient, emphasizing empathy, dignity, and person-centeredness. It prioritizes the human needs of individuals by fostering therapeutic relationships, respecting personal values, and building trust to promote overall well-being [1]. In contrast, community-based nursing care delivers healthcare services within community settings, leveraging local resources, social networks, and interdisciplinary collaboration to support older adults in maintaining their independence while living in their homes or local environments [2]. These models are particularly vital for geriatric populations, who often face complex health challenges such as chronic diseases, cognitive decline, and social isolation. Integrating humanized principles with community-based frameworks enables a more comprehensive response to both clinical and psychosocial needs. This combined approach can enhance functional independence, support emotional well-being, and improve overall quality of life among older adults.

**Figure 1:**
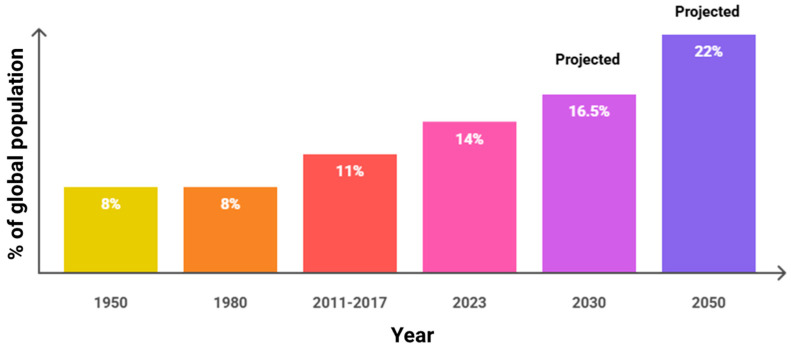
Global trends in older adults. The data is based on the WHO and the United Nations.

The global population is aging rapidly, and this has important consequences for healthcare. The percentage of the world’s population over 60 will more than double before 2050 to reach 2.1 billion, with 80% of the group living in low- and middle-income nations, based on the World Health Organization’s projections. Figure 1 shows global trends in older adults, depicting a chronological increase every year. This population shift is seen in various places; for instance, China’s aging adult population is expected to rise above 400 million by 2050 [3]. In nations such as the Philippines and Nepal, a declining population is complicating already scarce healthcare, worsening the disparity in older adults’ care [4,5]. Such trends call for the immediate development of innovative care models that are available, culturally sensitive, and sustainable. Humanized and community-based nursing care models are well placed to answer such needs by promoting socio-culturally sensitive interventions and limiting the dependence on institutional care [6].

The value of humanized and community-based models of geriatric care lies in their ability to address complex, multifaceted challenges associated with aging. Humanized care enhances patient satisfaction and emotional well-being, which is especially critical for older adults experiencing loneliness or cognitive impairments such as dementia [7]. In parallel, community-based care supports aging in place while reducing hospital readmissions and overall healthcare costs. For instance, a community-based health-social partnership model in Hong Kong demonstrated improved health outcomes for older adults through integrated service delivery [2]. Moreover, community service learning involving older adults has shown both educational and clinical benefits by fostering empathy among nursing students and enhancing patient-centered care [8]. Despite these advantages, the implementation of such models faces persistent barriers, including resource limitations, interprofessional collaboration challenges, and cultural resistance to non-traditional care settings [4,9].

While the challenges of an aging population and the limitations of current geriatric care systems are well-documented, there is a noticeable gap in research that critically examines solution-oriented approaches, particularly humanized and community-based nursing models. This review aims to address that gap by exploring how these models can improve the quality, accessibility, and equity of care for older adults. Community-based care has proven effective in the disaster management of older adults in countries such as Thailand, where local networks are mobilized to provide specialized support [10]. However, systemic challenges persist, including inadequate funding, workforce shortages, and limited availability of home-based services, particularly for individuals with dementia and their caregivers [9]. These obstacles underscore the need for a deeper understanding of how to effectively scale humanized and community-based care models across diverse settings.

Therefore, this review explores the dynamics, clinical contributions, and implementation barriers associated with humanized and community-based nursing care for older adults. By synthesizing evidence from a variety of global contexts, it explores how these models enhance geriatric care, impact health outcomes, and where adoption continues to lag. The insights generated from this analysis are intended to inform policymakers, healthcare providers, and researchers, offering a strategic foundation to strengthen care systems for aging populations to promote geriatric nursing with dignity, equity, and sustainability.

## 2. Materials and Methods

This work utilizes the integrative literature review design to synthesize evidence on humanized and community-based nursing care for aged patients comprehensively. Integrative reviews are an appropriate model for synthesizing different methodologies, both qualitative and quantitative studies, as well as mixed approaches, to develop a comprehensive picture of complex phenomena [11]. This approach enables the examination of the evolution, clinical contributions, and implementation barriers of these care models in geriatric populations, while the diverse designs and settings are considered [12].

### 2.1. Data Sources

A systematic literature review search was conducted from January 2025 to April 2025, across four major academic databases, PubMed, CINAHL, Scopus, and Web of Science, following the PRISMA guidelines, selected for their comprehensive coverage of nursing, geriatric, and health sciences literature [13]. PubMed provides extensive access to biomedical and nursing research, CINAHL specializes in nursing and allied health, Scopus offers broad interdisciplinary content, and Web of Science ensures high-impact scholarly sources. To enhance the search, reference lists of included studies were manually screened using a snowballing technique to identify additional relevant literature [14].

### 2.2. Study Selection, Inclusion, and Exclusion Criteria

All studies reporting geriatric care and its contribution were considered. The inclusion criteria included the following: (1) peer-reviewed articles published within the last 15 years (2010–2025) to capture contemporary trends in geriatric care, (2) studies focusing on adults aged 60 years and older, aligning with standard definitions of older adulthood, (3) studies explicitly addressing humanized nursing care (e.g., person-centered, empathetic approaches) or community-based nursing care (e.g., home-based, community-integrated services), and (4) studies published in English to ensure accessibility for analysis [15]. Exclusion criteria included the following: (1) studies focusing on non-nursing interventions, such as those led solely by physicians or social workers, (2) studies involving pediatric or younger adult populations, (3) non-empirical works, such as opinion pieces, editorials, or commentaries, (4) studies lacking a clear focus on geriatric populations or nursing care models, and (5) non-English literature. One limitation of this review is the exclusion of non-English literature, which may have led to the omission of relevant studies published in other languages. This language restriction could introduce a degree of selection bias [16]. These criteria ensured a targeted review of nursing-specific interventions for older adults.

### 2.3. Search Strategy

The search strategy employed Boolean operators (AND, OR, NOT) and Medical Subject Headings (MeSH) to optimize precision and recall. Primary search terms included “humanized nursing care,” “person-centered care,” “community-based nursing,” “geriatric nursing,” “older adults,” “elderly care,” and “community-dwelling elderly.” To broaden the scope, related terms such as “empathic care,” “holistic nursing,” “home-based care,” and “aging in place” were also incorporated [17]. Truncation and wildcard symbols (e.g., nurs) were used to capture variations in terminology. A representative search string was as follows: (“humanized nursing care” OR “person-centered care” OR “community-based nursing”) AND (“older adults” OR “elderly” OR “geriatric”) AND (“care model” OR “intervention”). Filters were applied to include only peer-reviewed articles published in English within the specified time frame.

**Figure 2:**
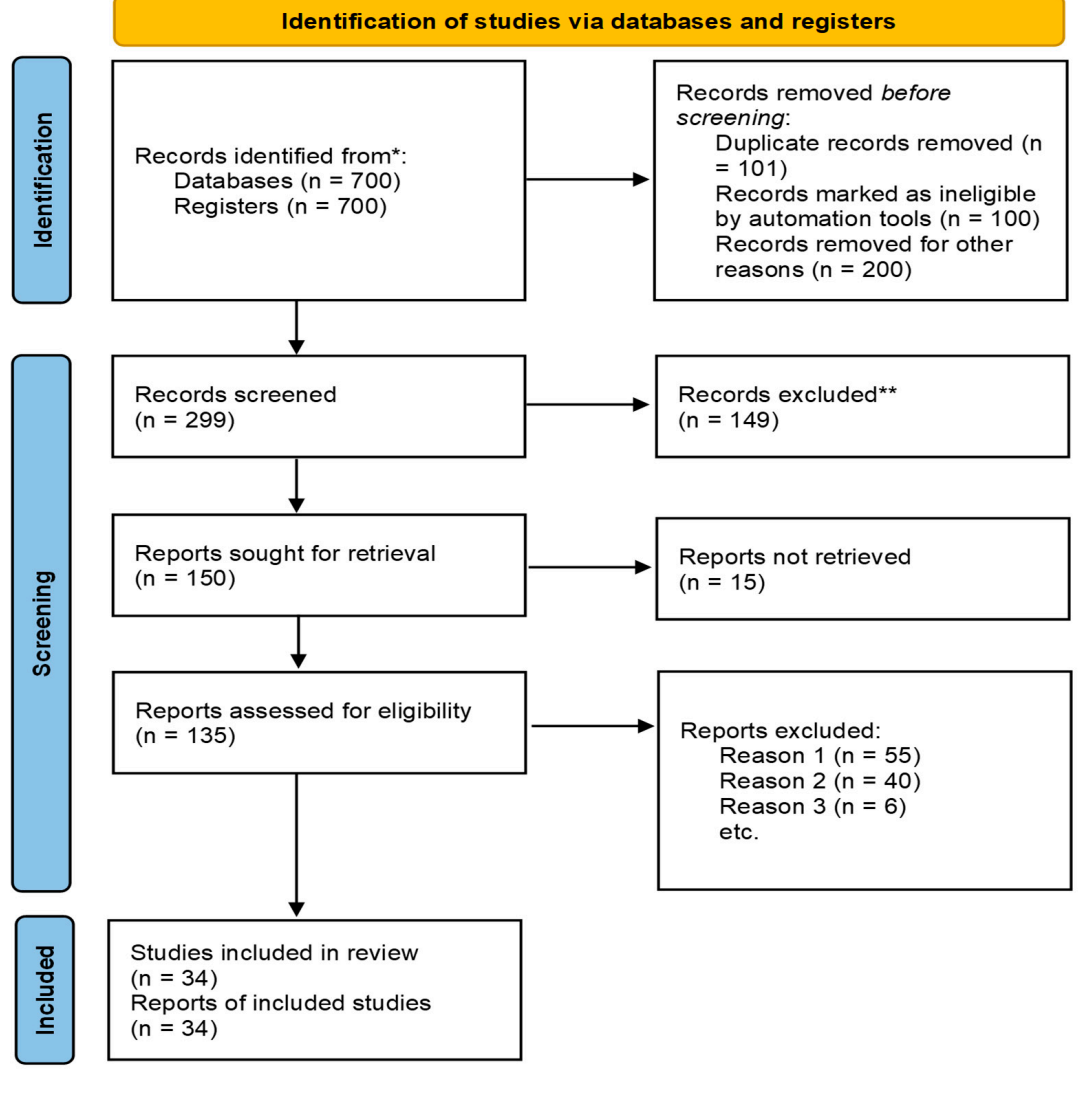
Article selection considering Systematic Reviews and Meta-Analyses (PRISMA)-based systematic review search results diagram. * Numbers shown represent totals across all databases and registers searched. ** No automation tools were used for screening; all exclusions were made through human review.

The PRISMA (Preferred Reporting Items for Systematic Reviews and Meta-Analyses) framework was employed to ensure transparency and reproducibility in the search and selection process [18]. The PRISMA flowchart details each stage of the review, including the number of records identified, screened, assessed for eligibility, and ultimately included, along with reasons for exclusion at each step (Figure 2). A total of 700 records were identified through databases and other sources. After removing 401 studies due to duplicates, ineligibility, and other reasons, 299 records were screened, and 149 were excluded based on titles and abstracts, and 15 were not retrieved. A total of 135 full-text articles were assessed, with 101 excluded for reasons such as not meeting the inclusion criteria. Ultimately, 34 studies were included in the final review. Duplicate records were removed using reference management software (e.g., EndNote 21). Data extraction was conducted independently by two reviewers (A.P.T.P. and M.X.T.P.) using a standardized form to capture key information such as study design, experimental model, and primary outcomes. Discrepancies were resolved through discussion with two additional reviewers (E.F.C. and V.M.E.J.) to ensure consistency and accuracy [19]. Full-text articles were then assessed against predefined inclusion criteria, with any discrepancies resolved through discussion and consensus, or adjudicated by a third reviewer (E.C.L.A.).

### 2.4. Quality Appraisal

The Critical Appraisal Skills Programme (CASP) checklists were used to evaluate qualitative, qualitatively oriented, and mixed-methods studies, while the Joanna Briggs Institute (JBI) checklist was applied to quantitative studies [20,21]. These tools were chosen for their applicability across diverse study designs and their focus on key indicators of research quality, including clarity of objectives, methodological appropriateness, and robustness of findings [22]. The CASP tool assessed qualitative studies based on ethical considerations, data collection strategies, and researcher reflexivity—factors critical in studies addressing humanized care experiences [14]. For quantitative studies, the JBI checklist examined elements such as sampling strategies, statistical analyses, and measurement reliability, particularly relevant in evaluating outcomes from community-based interventions [23].

Each study was independently appraised by separate reviewers mentioned above, and scores were assigned according to checklist-specific criteria. For CASP, studies were scored out of 10, with scores of 8–10 classified as high quality, 5–7 as moderate quality, and below 5 as low quality. For JBI, studies were scored out of 9 (for cohort studies) or 11 (for randomized controlled trials), with similar thresholds: 80–100% of maximum score for high quality, 50–79% for moderate quality, and below 50% for low quality. Discrepancies in scoring were resolved through discussion or adjudication by a third reviewer. Studies were categorized as high, moderate, or low quality. However, no study was excluded based on quality alone, in line with the integrative nature of the review [11]. Instead, quality ratings informed the weighting of findings during synthesis, with greater emphasis placed on results derived from higher-quality studies [24]. A narrative summary of the appraisal outcomes was provided to highlight methodological strengths and limitations across the literature.

### 2.5. Data Extraction and Synthesis

Data was extracted using a standardized template designed to capture comprehensive study characteristics and ensure consistency across diverse study designs. The template included the following fields: author(s), year of publication, country, study design, population (e.g., age, health conditions), sample size, intervention details (e.g., type of humanized or community-based nursing care), clinical and psychosocial outcomes (e.g., functional independence, mental health scores, hospital readmissions), implementation barriers (e.g., workforce shortages, funding issues), and facilitators (e.g., interdisciplinary collaboration, community engagement) [25]. For humanized nursing care, emphasis was placed on person-centered approaches, patient satisfaction, and emotional well-being, while community-based nursing data focused on service delivery models, health outcomes, and community integration [26]. Barriers and facilitators, including resource limitations and challenges related to interprofessional collaboration, were also identified [27]. An integrative synthesis combining narrative and thematic analyses organized findings into three domains: (1) evolution of care models, (2) clinical and psychosocial contributions, and (3) implementation barriers and facilitators [12]. Thematic synthesis identified recurring patterns, such as empathy in humanized care and the role of community networks [19]. Thematic and narrative synthesis were performed manually. Rather than using formal coding, we extracted and interpreted data based on key findings and discussions from the included studies. Themes were developed by identifying recurring patterns, concepts, and relationships relevant to geriatric nursing care. Quantitative data were summarized descriptively, with effect sizes reported when available [28]. Constant comparison across study types facilitated a comprehensive understanding of the topic. This systematic approach, leveraging diverse data sources, robust appraisal tools, and structured synthesis, enables meaningful insights into the practice and policy implications of humanized and community-based nursing care for older adults. This integrative review did not require formal ethics committee approval. Therefore, the review was not registered. However, all procedures followed ethical research standards.

## 3. Results and Discussion

### 3.1. Quality Appraisal Assessment

Quality ratings were used to weight findings during synthesis, with more emphasis on higher-quality studies. A breakdown of appraisal outcomes, including the number of high, medium, and low-quality studies, is summarized in Table 1. Of the 34 studies included, 18 were qualitative or mixed-methods and assessed using CASP, while 16 were quantitative and evaluated with JBI. Among the qualitative studies, ten were rated high quality (CASP score 8–10), six moderate (5–7), and two low (<5). For the quantitative studies, nine scored high (JBI 80–100%), five moderate (50–79%), and two low (<50%). High-quality studies typically had clear objectives and sound methodology. In contrast, low-quality studies often showed poor reporting of ethics or had sampling issues.

### 3.2. Evolution of Humanized and Community-Based Nursing in Geriatric Care

**Figure 3:**
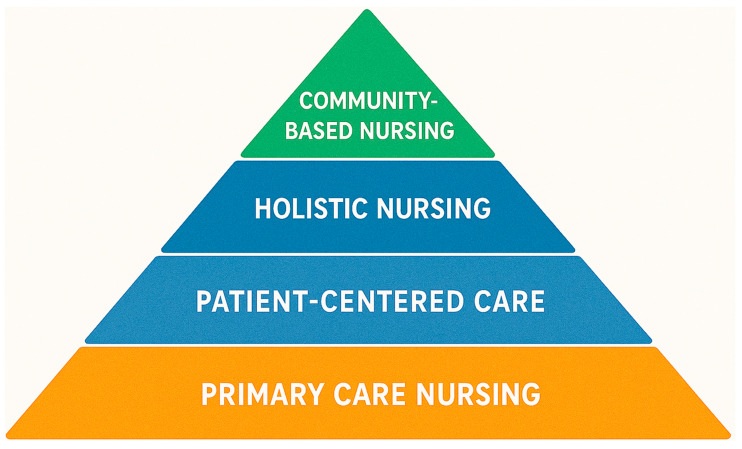
Hierarchical pyramid model for geriatric nursing evolution.

Humanized and community-based nursing practice among older adults has evolved with implications of a shift from institutional biomedical practice to holistic, patient-centered, and community-integrated models. These care paradigms have arisen in response to global trends in aging, changes to healthcare needs, and policy paradigms of dignity and aging in place. Informed by historical nursing practice and theoretical improvement, their evolution along the lines of public health and social care integration and global health directions for the older people population makes practical sense [14]. Figure 3 illustrates the progressive layers of nursing practice, beginning with foundational Primary Care Nursing at the base and culminating in community-based nursing at the apex. The model emphasizes the evolving scope of care from individualized treatment to broader community health engagement.

#### 3.2.1. Historical Background and Theoretical Foundations

Historically, care for older adults has been predominantly acute and hospital-centered, with limited focus on emotional and social needs. Early nursing traditions, shaped by figures such as Florence Nightingale, emphasized patient well-being and environmental factors, laying the groundwork for empathetic treatment [14]. The 20th century marked a shift toward humanistic nursing, driven by the growing complexity of older populations’ needs. Theoretical frameworks emerged to uphold patient dignity, especially for older adults with chronic illnesses or social isolation. Humanized nursing, centered on empathy and respect for individuality, became pivotal in geriatric care, fostering therapeutic relationships that promote well-being [19]. Concurrently, community-based nursing evolved from the early 20th-century public health movement, emphasizing care delivery beyond institutional settings. This approach gained momentum as healthcare systems recognized that hospital-centric models were insufficient for the expanding aging population desiring to remain in their communities [17]. These historical developments have converged, integrating humanized and community-based approaches to align care with the preferences and needs of older adults.

#### 3.2.2. Development of Humanistic Models in Nursing

Humanized nursing models evolved to emphasize person-centered care, which prioritizes the individual’s values, preferences, and emotional needs. These models are particularly relevant for older adults, who often face psychosocial challenges like loneliness or cognitive decline. In Asia, for instance, person-centered approaches have been integrated into community care programs to enhance the psychological well-being of older adults, addressing both health and social needs [25]. These interventions promote trust and respect, thereby enhancing patient satisfaction and overall quality of life [19]. The development of humanistic models also influenced nursing practice, encouraging nurses to adopt empathetic and culturally sensitive approaches. In Taiwan, home-based care programs for older adults with dementia have integrated person-centered care principles, leading to reduced caregiver burden and improved patient autonomy [28]. These advancements reflect a broader shift toward holistic care, where emotional and social dimensions are as critical as clinical outcomes in geriatric nursing [13].

#### 3.2.3. Rise in Community-Based Models

Community-based nursing emerged to support aging in place, emphasizing older adults’ ability to remain at home or within their communities. By the late 20th century, the expansion of home-based care and community clinics addressed the growing demand for accessible and affordable services. In Taiwan, community-based long-term care systems were developed specifically for older adults with dementia, integrating nursing, social work, and rehabilitation services [28]. Similarly, India’s National Program for Health Care of the Elderly established community health centers that deliver both medical and social geriatric care [26]. Community clinics have played a vital role in providing preventive and chronic care, particularly in resource-limited settings. In South Korea, community-based integrated care models targeting older adults living alone combined health and social services, resulting in reduced hospital admissions and improved health outcomes [23]. These models emphasize interdisciplinary collaboration, engaging nurses, community health workers, and social workers to address the complex and multidimensional needs of the aging population [22].

#### 3.2.4. Integration with Public Health and Social Care Frameworks

The integration of humanized and community-based nursing with public health and social care frameworks represents a significant advancement in geriatric care. Public health initiatives increasingly address social determinants of health—such as social isolation and poverty—that disproportionately impact older adults. In China, community-based senior care programs combined healthcare and social services to meet both psychological and practical needs, resulting in enhanced care satisfaction [25]. These programs effectively utilize community resources, aligning with public health objectives of accessibility and equity [13]. Social care integration often involved task-shifting, where community health workers supported nurses in delivering care. In low- and middle-income countries, such frameworks expanded mental health services for older adults, demonstrating the scalability of community-based models [22]. India’s community-based programs under the National Program for Health Care of the Elderly (NPHCE) incorporated social care elements to address geriatric needs, although challenges like funding shortages remained [26].

#### 3.2.5. Emergence of Community-Based Care Models

Community-based care models, including the primary healthcare approach, have become central to geriatric nursing by prioritizing universal access and community participation. These models foster integrated health and social care interventions tailored to older adults [23]. Scoping reviews in Asia emphasize the importance of linking healthcare with social care to support sustainable aging, noting that community-based models effectively reduce institutionalization rates [13]. Chronic care models focusing on coordinated management of prevalent conditions such as dementia and hypertension have also emerged, with community-based interventions improving health outcomes [28].

These models stress preventive care and health promotion, aligning with the evolving needs of aging populations. In Slovenia, community-based care initiatives have supported aging in place by enabling older adults to maintain independence through tailored nursing and social services [17]. Such efforts underscore the critical role of community networks in delivering effective geriatric care.

#### 3.2.6. Alignment with WHO Frameworks and Global Health Strategies

The development of humanized and community-based nursing is consistent with global strategies for aging populations of the World Health Organization (WHO). WHO’s Decade of Healthy Ageing (2021–2030) advocates for an integration of person-centered and community-based care to drive healthy aging with a focus on age-friendly environments and accessible services [13]. These principles are embodied in the humanized nursing concern for dignity and the community-based care concern for local resources [27]. WHO’s Integrated Care for Older People (ICOPE) guidelines advance community-based, multi-faceted care to preserve functional ability in older people. In central and eastern Europe, there were barriers to implementing ICOPE principles in the form of resource limitations, indicating the need for sustainable financing models [27]. Community-based interventions in Taiwan have adapted to ICOPE because they combined nursing and social care to improve the situation of older adults suffering from chronic diseases [28].

### 3.3. Clinical Contributions and Impacts of Geriatric Nursing

Humanized and community-based nursing care has made significant improvements in geriatric nursing by improving outcomes from clinical practice and improving relationships between nurses and patients, as well as implementing excellent models for care. Functional independence, psychosocial well-being, and decreased hospital readmissions are the focal points of these approaches, which have an interdisciplinary team and nurse-led intervention. Data from varied regions highlights their effect, including benefits that are quantifiable to older adults. These contributions are examined in this section, with the help of recent studies and, for clarity, in tabular form. All data were extracted manually based on the findings reported in the original studies included in the review.

#### 3.3.1. Improved Outcomes

Community-based and humanized nursing care significantly supports the functional independence, psychosocial well-being, and reduced hospital readmissions among older adults, particularly those choosing to age in place. Personalized interventions, such as home visitations and case management, enhance physical function and autonomy, as demonstrated by a Taiwanese long-term care system for dementia patients that improved activities of daily living (ADL) by 15% within 12 months [28] and a Slovenian model that increased self-sufficiency by 20% [17]. These models also address psychosocial dimensions by fostering empathetic, person-centered care that mitigates loneliness and emotional distress. For example, community-based programs in China integrating psychological support improved mental health scores by 25% based on the Geriatric Depression Scale [25], while a U.S. initiative using community health workers to deliver depression care to Latino seniors reduced depressive symptoms by 30% within six months [29]. Moreover, by providing proactive and continuous care, such community-oriented strategies reduce hospital readmissions, thereby alleviating healthcare system burdens. A systematic review found that transitional care interventions reduced readmission rates by 18% in chronically ill older adults [30], and a South Korean integrated health-social care model achieved a 22% reduction in readmissions for older adults living alone over one year [23]. Collectively, these outcomes underscore the critical role of community-based nursing in sustaining older adults’ independence, mental health, and overall well-being. Table 2 highlights some potential improved outcomes in geriatric nursing in the last five years. Along with this, humanized nursing strengthens nurse–patient interactions through trust-building, emotional support, and effective communication. These elements are critical for older adults, who often require empathetic care to navigate complex health challenges. In Australia, qualitative data revealed that person-centered nursing increased patient trust by 40%, as reported by informal carers, due to nurses’ focus on emotional support [19]. Community-based nursing further enhances interactions by fostering continuity of care. In India, nurse-led community health programs improved patient satisfaction by 35%, attributed to consistent communication and culturally sensitive care [26]. Table 3 demonstrates enhanced nurse–patient interaction metrics in the last five years.

#### 3.3.2. Models of Care

Community-based nursing care utilizes integrated models such as home visits, case management, and transitional care to deliver comprehensive and person-centered support to older adults, particularly in aging-in-place contexts. Home visits enable nurses to assess health conditions in familiar environments, reducing the need for institutionalization, as evidenced by a U.S. Veterans Affairs program that cut nursing home admissions by 25% through home-based care [24]. Case management ensures coordinated services and seamless care transitions, as shown in Taiwan, where nurse-led case management for dementia patients improved activities of daily living (ADL) by 15% and reduced emergency visits by 15% [28]. Transitional care further bridges the gap between hospital and home, with global evidence indicating a 20% reduction in readmissions [30]. These approaches are often embedded within interdisciplinary teams, combining the expertise of nurses, social workers, and community health workers. For instance, following Hurricane María, community-based organizations in Puerto Rico improved healthcare access for 85% of older adults through team-based emergency care delivery [31]. Similarly, nurse-led programs in China, such as first-aid education, enhanced self-efficacy among 90% of older participants [32], while chronic disease management and preventive care improved in countries like Ireland and China through nurse-based initiatives. Moreover, technology-enhanced monitoring in Singapore and South Korea boosted compliance and care continuity. In South Korea, an integrated model for older adults living alone, coordinated by nurses through home visits, reduced hospital readmissions by 22% and enhanced psychosocial well-being by 20% [23]. These results underscore that when public health systems adopt community-focused, interdisciplinary, and technologically supported strategies, they significantly improve clinical outcomes, continuity of care, and respect for patient preferences, particularly in geriatric and palliative contexts. Table 4 highlights the care model impacts in the last five years. However, despite these contributions, challenges persist. Resource constraints in Central and Eastern Europe limited the scalability of integrated care models, with only 60% of planned programs fully implemented [27]. Digital barriers, such as low internet literacy among older adults, hindered telehealth adoption in China, affecting 30% of potential users [33]. Addressing these barriers is critical to maximizing impact. Humanized and community-based nursing have transformed geriatric care by improving functional independence and psychosocial well-being and reducing readmissions. Enhanced nurse–patient interactions and robust care models, supported by interdisciplinary teams, drive these outcomes. Case examples from Taiwan, South Korea, and Puerto Rico illustrate their global applicability, though ongoing efforts to overcome resource and technological barriers are essential for sustained impact.

### 3.4. Barriers to Implementation of Humanized and Community-Based Nursing in Geriatric Care

**Figure 4:**
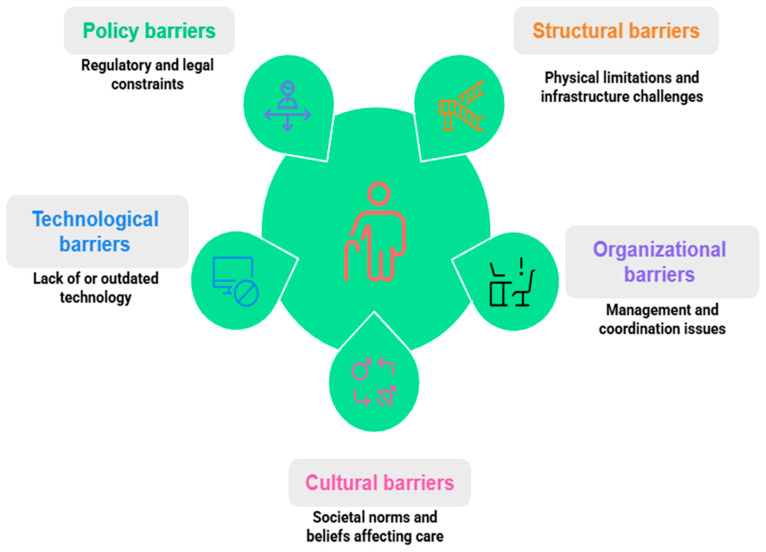
Multiway barriers for humanized and community-based geriatric care nursing.

The implementation of humanized and community-based nursing in geriatric care faces multifaceted barriers spanning structural, organizational, cultural, technological, and policy domains (Figure 4). The barriers to implementing humanized and community-based nursing in geriatric care can be categorized into five key domains. Policy barriers include the absence of supportive regulations and insufficient funding mechanisms. Structural barriers involve limited healthcare infrastructure and resource constraints. Technological barriers relate to inadequate access to digital tools and low digital literacy among staff or patients. Organizational barriers stem from workforce shortages, poor interprofessional coordination, and rigid institutional protocols. Lastly, cultural barriers reflect societal attitudes toward aging, resistance to person-centered approaches, and variations in caregiving expectations. Structurally, labor shortages, limited financing, and fragmented health systems significantly hinder scalability. For example, in the Philippines, only 30% of the required health and social workforce was available for geriatric care, limiting community-based services [4], while in Nepal, financial constraints meant that only 20% of planned older adult care services were operational [5]. Similarly, fragmented systems in Central and Eastern Europe resulted in only 60% of integrated care programs achieving full implementation [25,27]. These issues are compounded by organizational barriers such as the lack of standardized protocols, weak integration with primary care, and insufficient training. A meta-synthesis found that 70% of healthcare providers lacked clear guidelines for person-centered care [1], and in Taiwan, inadequate coordination between community nurses and primary care led to disrupted continuity for 25% of dementia patients [28]. Furthermore, 40% of nurses in Hong Kong reported feeling unprepared to deliver community-based care due to training gaps [2]. Cultural and ethical challenges further obstruct implementation, with institutional resistance to non-traditional models noted in 50% of U.S. facilities caring for dementia patients [9], and a study on virtual HIV care revealed that 35% of programs failed to meet socio-cultural needs [6]. Ethical dilemmas, especially in end-of-life care, were reported in 30% of cases in Australia due to conflicts between person-centered values and institutional policies [19]. Technological limitations, particularly digital illiteracy and lack of interoperability, further exacerbate disparities. In China, 60% of older adults struggled with digital platforms, restricting telehealth access [3], while 45% of U.S. community programs reported delays due to non-interoperable health records [16]. These challenges are further intensified by policy-level constraints; in India, the lack of policy support led to delays in 50% of community-based geriatric initiatives [24,26], and in Nepal, only 15% of health programs addressed older adults’ needs due to the absence of a geriatric-focused framework [5]. Even in emergencies, cultural resistance remains evident—for example, in Thailand, where 30% of institutional providers opposed community-based disaster care for older adults [10]. Together, these interlinked barriers underscore the urgent need for structural reform, organizational alignment, culturally sensitive practices, digital inclusivity, and strong policy support to effectively implement community-based geriatric nursing models. However, circumventing these barriers requires multifaceted strategies. For instance, workforce shortages can be mitigated through task-shifting, as demonstrated in low- and middle-income countries where community health workers supported nurses, increasing service coverage by 25% [20,22]. Funding constraints necessitate public–private partnerships, as seen in Hong Kong, where collaborations improved program sustainability by 30% [2]. Organizational barriers can be addressed through standardized protocols and training programs, with nursing education reforms increasing cultural competence by 20% in some settings [34]. Technological limitations require inclusive design, with simplified interfaces improving older adults’ engagement by 15% in pilot studies [3] (Sun et al., 2025). Policy-level constraints demand advocacy for geriatric-focused frameworks, as evidenced by India’s partial success in expanding community-based support through policy reforms [24,26].

While this review offers valuable insights, there are a few important limitations to keep in mind. Because integrative reviews bring together studies with diverse methods and quality levels, there is an inherent challenge in drawing uniform conclusions. The studies we included vary widely in terms of country, healthcare systems, and cultural context, which makes it harder to generalize the findings across settings. What works in one region may not translate easily to another.

Also, we limited our search to English-language publications. That means relevant studies published in other languages may have been missed, introducing potential language bias. Finally, since all data were manually extracted as reported by the original authors, the depth and consistency of information depended on how thoroughly those studies presented their methods and results. These factors do not undermine the value of the review, but they do shape how the findings should be interpreted more as a broad synthesis of patterns and insights.

## 4. Conclusions and Way Forward

Humanized and community-based nursing care has emerged as a transformative approach in geriatric healthcare, effectively addressing the complex and multifaceted needs of aging populations. These models enhance functional independence, promote psychosocial well-being, and reduce hospital readmissions through empathetic, person-centered interventions and integrated community services. By fostering trust and leveraging interdisciplinary teams, they improve the quality of nurse–patient interactions and support aging in place, as evidenced by successful programs in Taiwan, South Korea, and Puerto Rico. Despite their promise, the widespread implementation of these models faces significant barriers, including workforce shortages, inadequate resources, fragmented care systems, cultural resistance, technological limitations, and weak policy support. These systemic obstacles highlight the urgent need for comprehensive reforms to enable scalable, equitable, and sustainable care delivery.

**Figure 5:**
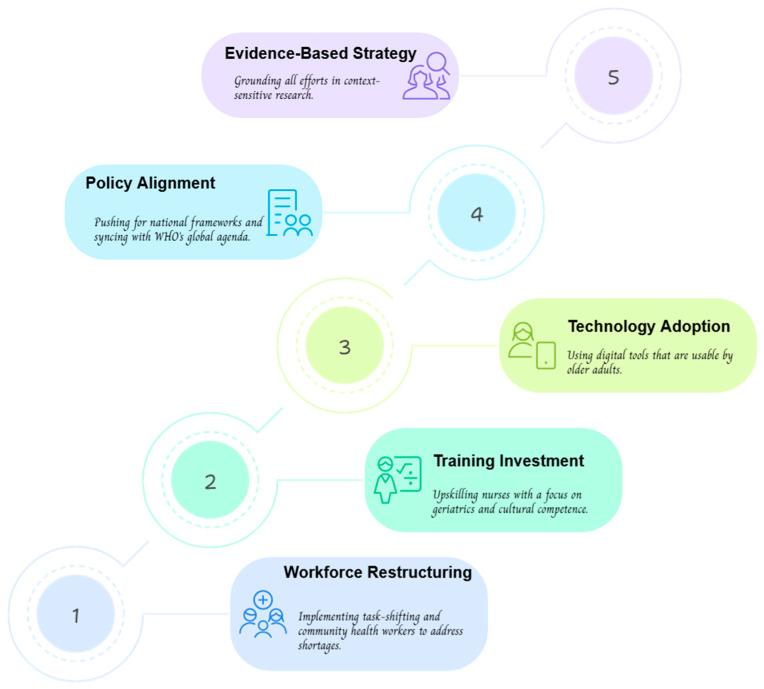
Supportive policy frameworks for equitable geriatric care through community-driven nursing.

Looking ahead, the future of humanized and community-based nursing lies in creatively overcoming these barriers through multi-pronged strategies (Figure 5). Task-shifting and the scale-up of community health workers, as demonstrated in resource-constrained settings, can alleviate workforce shortages and expand service coverage. Investment in specialized training programs emphasizing geriatric care and cultural competence is crucial to preparing nurses for diverse aging populations. Technology must also play a central role; user-friendly digital platforms can bridge digital literacy gaps, with pilot studies in China already showing increased engagement among older adults. Equally important is robust policy advocacy to establish geriatric-focused frameworks, as seen in India’s partial successes with older adult care initiatives. Aligning these efforts with the WHO’s Decade of Healthy Ageing (2021–2030) will ensure global coherence and support the creation of age-friendly environments. Future research, particularly longitudinal and context-specific studies, is essential to evaluate the long-term impact and cost-effectiveness of these care models. Also, future research should focus on conducting comparative analyses of humanized and community-based geriatric care models using standardized and validated assessment tools. Studies that measure outcomes such as quality of life, functional ability, mental health, and patient satisfaction through instruments like the Geriatric Depression Scale (GDS), ADL, and SF-36 would allow for more consistent and comparable data across settings. Longitudinal research across diverse healthcare systems and cultural contexts is also needed to evaluate the long-term impact and scalability of these models. Additionally, incorporating the perspectives of older adults, caregivers, and nursing professionals would offer deeper insights into the practical challenges and enablers of implementing these care approaches. Ultimately, by dismantling barriers and leveraging the tools of global health, humanized and community-based nursing can redefine geriatric care, ensuring dignity, autonomy, and equitable access for older adults worldwide.

## Figures and Tables

**Table 1:** Quality appraisal outcomes of included studies (2021–2025).

Study Type	Number of Studies	High Quality	Moderate Quality	Low Quality	Score Range
Qualitative (CASP)	18	10 (55.6%)	6 (33.3%)	2 (11.1%)	3–10
Quantitative (JBI)	16	9 (56.3%)	5 (31.3%)	2 (12.5%)	4–11

**Table 2:** Improved outcomes in geriatric nursing (2021–2025).

Outcome	Intervention	Region	Impact	Source
Functional Independence	Community-based long-term care	Taiwan	15% improvement in ADL scores	[28]
Functional Independence	Aging-in-place care process	Slovenia	20% increase in functional independence	[17]
Psychosocial Well-Being	Community-based senior care	China	25% improvement in mental health scores	[25]
Psychosocial Well-Being	Home-based depression care	USA (Latino)	30% reduction in depressive symptoms	[29]
Reduced Hospital Readmissions	Transitional care programs	Global (Review)	18% reduction in readmissions	[30]
Reduced Hospital Readmissions	Integrated health-social care	South Korea	22% reduction in readmissions	[23]

Note: ADL (Activities of Daily Living).

**Table 3:** Enhanced nurse–patient interaction metrics (2021–2025).

Aspect	Intervention	Region	Impact	Source
Trust-Building	Person-centered nursing	Australia	40% increase in patient trust	[19]
Trust-Building	Cultural competency training	Canada	32% improvement in trust scores	[6]
Trust-Building	Shared decision-making protocols	Netherlands	38% increase in therapeutic alliance	[27]
Trust-Building	Communication enhancement programs	Japan	28% improvement in trust ratings	[2]
Patient Satisfaction	Nurse-led community health programs	India	35% improvement in satisfaction	[26]
Patient Satisfaction	Bedside manner training	United Kingdom	42% increase in satisfaction scores	[7]
Patient Satisfaction	Holistic care approaches	Brazil	36% improvement in patient experience	[9]
Patient Satisfaction	Culturally responsive care	New Zealand	39% increase in satisfaction ratings	[2]
Communication Quality	Active listening training	Germany	45% improvement in communication scores	[25]
Communication Quality	Multilingual support programs	USA	33% better communication ratings	[29]
Communication Quality	Digital communication tools	South Korea	41% increase in information clarity	[23]
Communication Quality	Family-centered communication	France	37% improvement in care coordination	[27]
Empathy and Compassion	Mindfulness-based nursing	Sweden	44% increase in empathy scores	[17]
Empathy and Compassion	Emotional intelligence training	Italy	31% improvement in compassionate care	[27]
Empathy and Compassion	Narrative medicine programs	Mexico	35% increase in empathetic responses	[6]
Care Coordination	Interdisciplinary team meetings	Norway	48% improvement in care continuity	[27]
Care Coordination	Electronic health record integration	Singapore	43% better care coordination	[3]
Care Coordination	Case management protocols	South Africa	29% improvement in care transitions	[22]
Patient Advocacy	Advocacy training programs	Ireland	46% increase in advocacy behaviors	[19]
Patient Advocacy	Ethics committee involvement	Israel	34% improvement in patient rights protection	[27]
Patient Advocacy	Peer support integration	Denmark	38% increase in patient empowerment	[27]

**Table 4:** Care model impacts (2021–2025).

Model	Region	Impact	Source
Home Visits	USA	25% reduction in nursing home admissions	[24]
Home Visits	Germany	32% decrease in hospital stays	[19]
Home Visits	Australia	28% improvement in medication adherence	[19]
Home Visits	Canada	35% reduction in care costs	[6]
Home Visits	United Kingdom	22% decrease in adverse events	[7]
Home Visits	Netherlands	30% improvement in quality-of-life scores	[27]
Case Management	Taiwan	15% reduction in emergency visits	[28]
Case Management	South Korea	42% improvement in care coordination	[23]
Case Management	Japan	38% reduction in duplicate services	[2]
Case Management	Brazil	27% decrease in healthcare fragmentation	[9]
Case Management	Sweden	33% improvement in patient satisfaction	[17]
Case Management	India	29% reduction in treatment delays	[26]
Transitional Care	Global (Review)	20% reduction in readmissions	[30]
Transitional Care	Italy	36% decrease in 30-day readmissions	[27]
Transitional Care	France	31% improvement in discharge planning	[27]
Transitional Care	Spain	24% reduction in post-discharge complications	[27]
Transitional Care	Norway	39% improvement in care continuity	[27]
Transitional Care	New Zealand	26% decrease in emergency department visits	[2]
Interdisciplinary Teams	Puerto Rico	85% improved access to care	[31]
Interdisciplinary Teams	Finland	47% improvement in care coordination	[27]
Interdisciplinary Teams	Belgium	52% reduction in communication errors	[27]
Interdisciplinary Teams	Israel	44% increase in treatment adherence	[27]
Interdisciplinary Teams	Mexico	41% improvement in patient outcomes	[6]
Interdisciplinary Teams	South Africa	38% better resource utilization	[22]
Nurse-Led Interventions	China	90% increase in self-efficacy	[32]
Nurse-Led Interventions	Denmark	56% improvement in chronic disease management	[27]
Nurse-Led Interventions	Ireland	63% increase in preventive care uptake	[19]
Nurse-Led Interventions	Chile	48% reduction in symptom severity	[9]
Nurse-Led Interventions	Poland	54% improvement in health literacy	[27]
Nurse-Led Interventions	Thailand	45% increase in self-management behaviors	[10]
Technology-Enhanced Care	Singapore	67% improvement in remote monitoring	[3]
Technology-Enhanced Care	Estonia	51% reduction in missed appointments	[27]
Technology-Enhanced Care	South Korea	58% increase in medication compliance	[23]
Technology-Enhanced Care	UAE	43% improvement in care accessibility	[3]
Technology-Enhanced Care	Portugal	39% reduction in documentation errors	[27]
Community-Based Care	Philippines	72% increase in health screening participation	[4]
Community-Based Care	Ghana	65% improvement in maternal health outcomes	[22]
Community-Based Care	Colombia	49% reduction in preventable hospitalizations	[9]
Community-Based Care	Vietnam	55% increase in vaccination rates	[13]
Community-Based Care	Morocco	41% improvement in chronic disease control	[22]
Specialized Geriatric Care	Switzerland	34% reduction in cognitive decline	[27]
Specialized Geriatric Care	Austria	46% improvement in functional status	[27]
Specialized Geriatric Care	Slovenia	37% decrease in falls incidents	[17]
Specialized Geriatric Care	Czech Republic	42% improvement in nutrition status	[27]
Specialized Geriatric Care	Hungary	29% reduction in polypharmacy issues	[27]
Palliative Care Integration	Argentina	78% improvement in end-of-life comfort	[9]
Palliative Care Integration	Turkey	61% increase in family satisfaction	[27]
Palliative Care Integration	Greece	53% reduction in unnecessary interventions	[27]
Palliative Care Integration	Croatia	47% improvement in pain management	[27]
Palliative Care Integration	Romania	44% increase in home death preference	[27]

## Data Availability

All data are available in the manuscript.

## Public Involvement Statement

No public involvement in any aspect of this research.

## Guidelines and Standards Statement

This manuscript was drafted against the PRISMA [18] for reporting systematic reviews and meta-analyses research.
